# Supplementary material for: Revolutionizing Molecular cloning: Introducing FastCloneAssist, a Streamlined Python tool for optimizing primer design in restriction & ligation-independent PCR cloning
Source: PLoS One. 2025 Mar 13;20(3):e0306950. doi: 10.1371/journal.pone.0306950 (PMC11906075; doi:10.1371/journal.pone.0306950)
Supplement: S3 File — (DOCX) [file pone.0306950.s003.docx]

**Step-by-step protocol to use the FastCloneAssist in local Python Environment** Download the script file (S3) and open it in your choice of python environment. Here, shown in Microsoft Visual Studio integrated development environment but can be run in other environments too.

The script has the following steps to follow.

**Step1:** Install required libraries. Run the first part (Libraries installation) of the script. It will take a few minutes once it is done move to the next step. Note, you only need to do this one time for a computer machine.


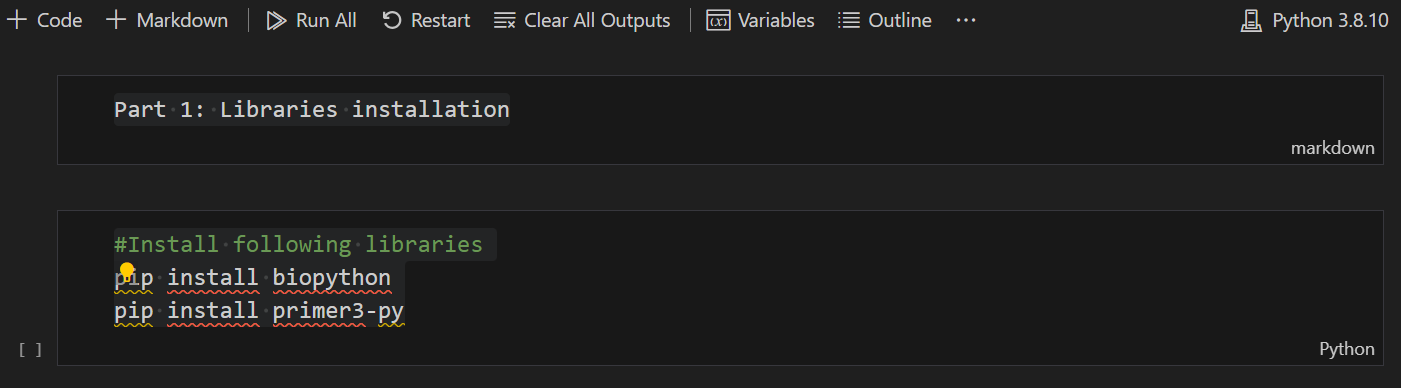


**Step 2:** Run the next part (Import Modules), it will import the required modules from above installed libraries, need to run each time once you after re-start of the system.


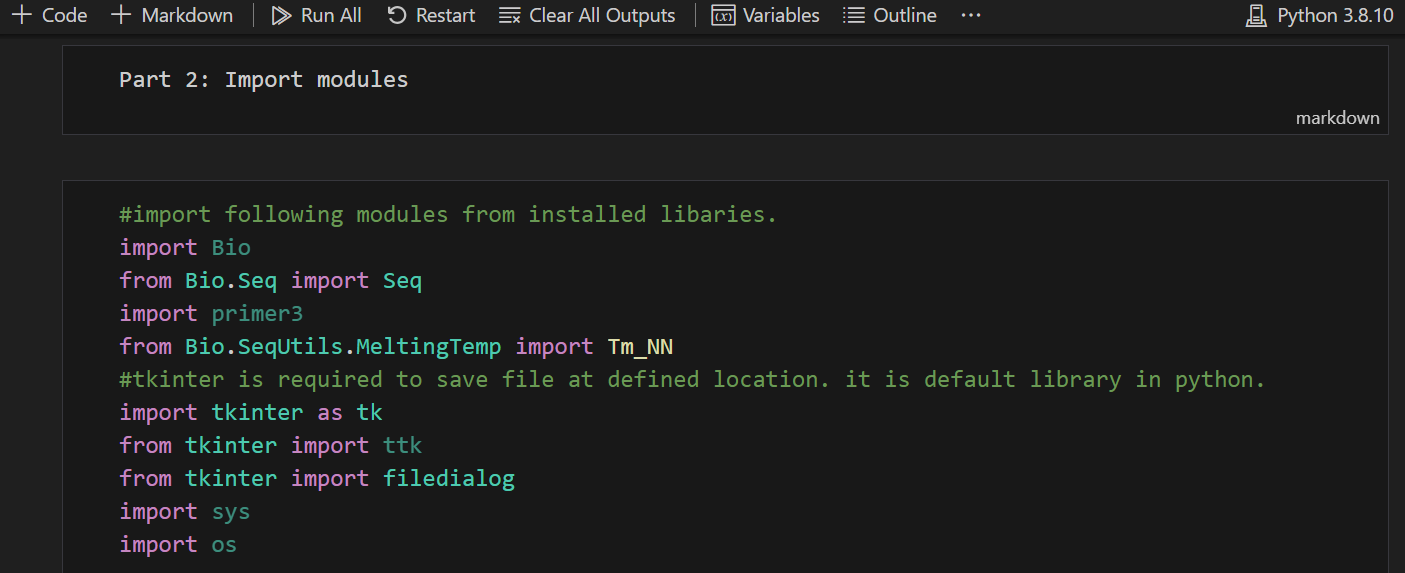


**Step3:** Design your primers. Run the third part (Primer Design) and follow instructions there.


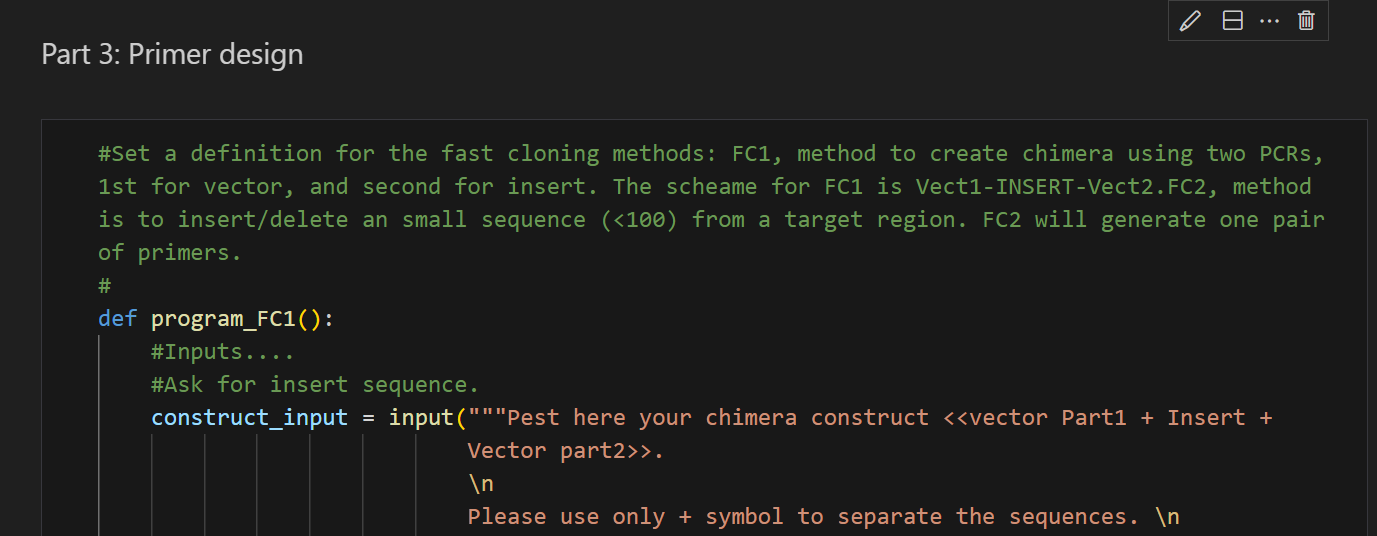


Once you run this part of script it will ask to choose the method (Class) of primer design.

**Sub step 3a:** Now the program asks to input your choice to select fast clone class, 1 or 2. The Classes are defined in the manuscript, please make your input sequence as described in the manuscript and Supporting document file S5.


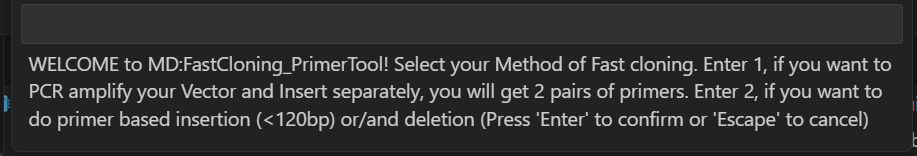


Here are the steps described using 1, add 1 and press enter.

**Sub step 3b:** Provide your sequence in the requested format, see the manuscript for more details.


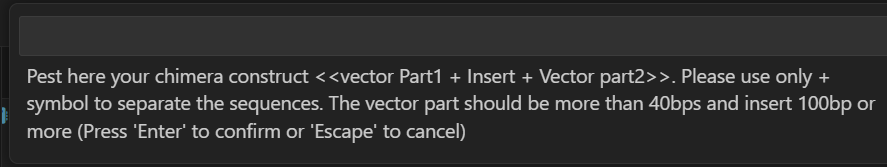


Press enter.

**Sub step 3c:** Next option will ask to choose required Tm in a range format.

Do you want to provide specific range of Tm, Yes or No.

If “No” it moves to next step and if “Yes”, it will ask for Tm.

If you input yes, add Tm range in the next step.


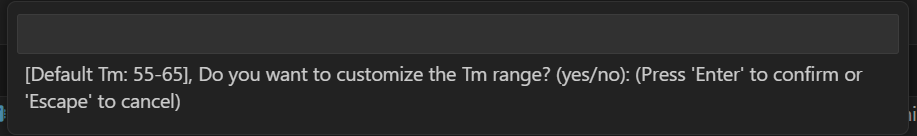


**Sub step 3d: Save the designed primer as results in a text fil.**

You will see the next windows open up to ask to save your results.

Note: Please minimize the python screen windows if the save page is not visible.


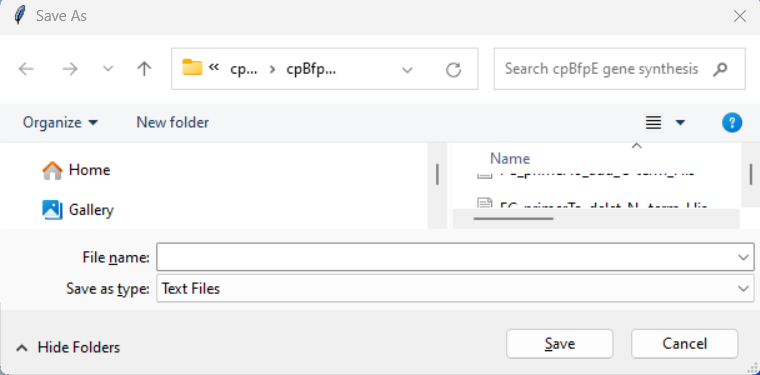


Your results will be saved as Text file at your defined locations.

**See example results in supplementary file S6 and S7.**

**Similar steps will be needed to follow for FC_class 2 primer designing, but the sequence input format will be different. See the manuscript for the details.**
