# Supplementary material for: Revolutionizing Molecular cloning: Introducing FastCloneAssist, a Streamlined Python tool for optimizing primer design in restriction & ligation-independent PCR cloning
Source: PLoS One. 2025 Mar 13;20(3):e0306950. doi: 10.1371/journal.pone.0306950 (PMC11906075; doi:10.1371/journal.pone.0306950)
Supplement: S4 File — (DOCX) [file pone.0306950.s004.docx]

**Step-by-step protocol to use the FastCloneAssist in Google Colab environment Step 1:** Download the FastCloneAssist script file for Google Colab (S4) from the supplementary files or from the Github link.

https://github.com/ps-vcu/FastCloneAssist.git

**Step 2: If you do not have a Google Colab account** create it using a Gmail account, see “https://colab.research.google.com” website for detail.

Now open the FastCloneAssist_Colab in your Google Colab.

**Step 3:** Libraries Installation, run this part by clicking the triangle and wait. You should see as the screenshot copied below.


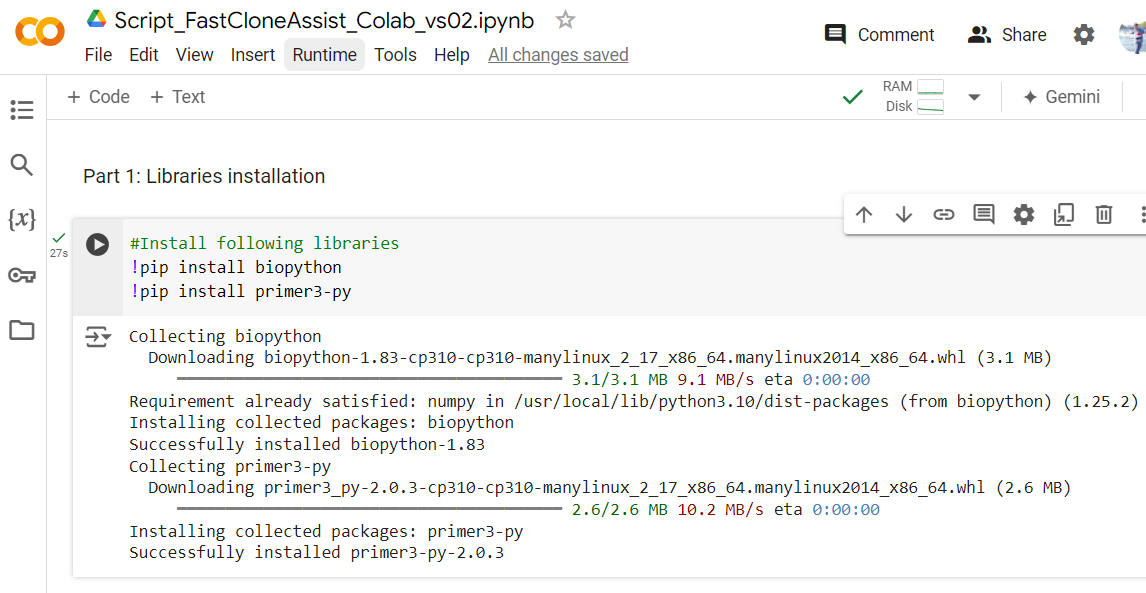


**Step 4:** Now run the next part (Import Modules), it will import the required modules from above installed libraries, need to run each time once you after re-start of the system.


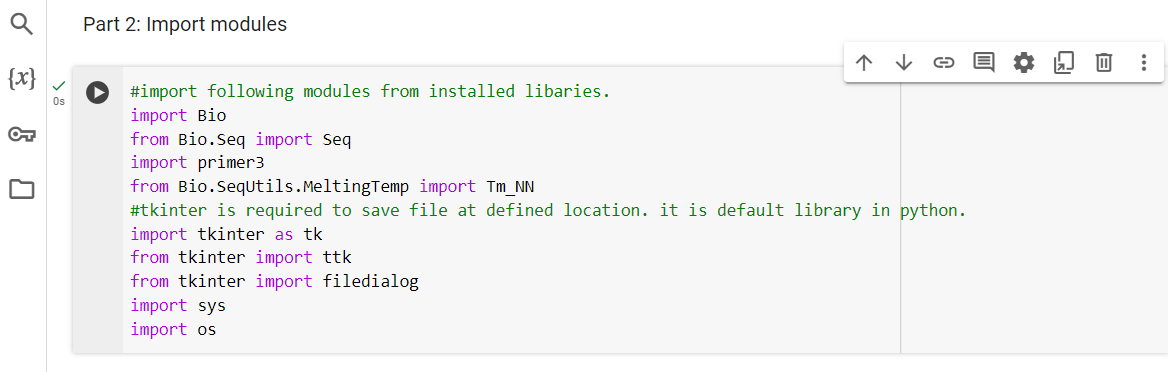


**Step 5:** Design your primers. Run the third part (Primer Design) and follow instructions there.

Once you run this part of script it will ask to choose the method (Class) of primer design.

**Sub step 5a:** Now the program ask to input your choice to select fast clone class, 1 or 2. The Classes are defined in the manuscript, please make your input sequence as described in the manuscript and in supporting file S5.


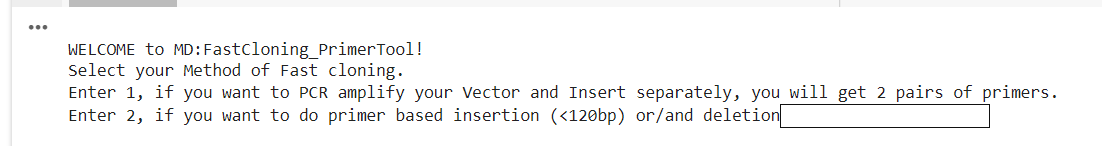


Here are the steps described using 1, add 1 and press enter.

**Sub step 5b:** Provide your sequence in the requested format, see the manuscript for more details.


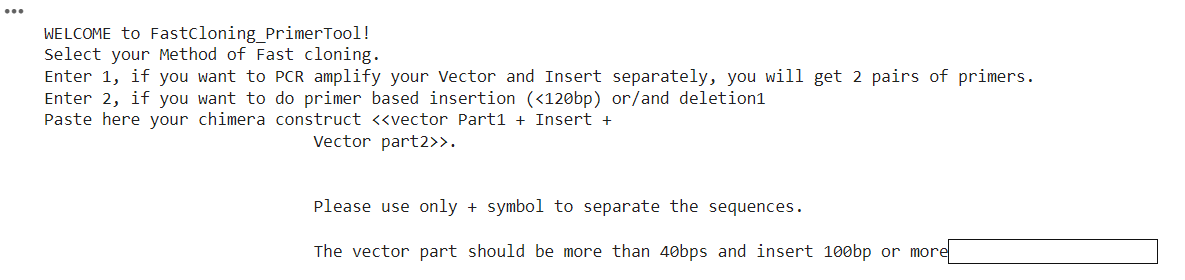


Press enter.

**Sub step 5c:** Next option will ask to choose required Tm in a range format.

Do you want to provide a specific range of Tm, Yes or No.

If “No” it moves to next step and if “Yes”, it will ask for Tm.

If you input yes, add Tm range in the next step.


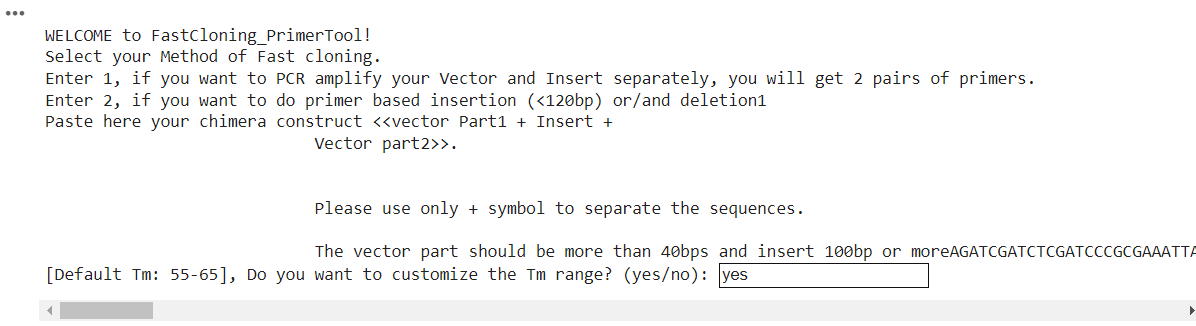


If the input is yes, net popup will ask to add the Tm rang.


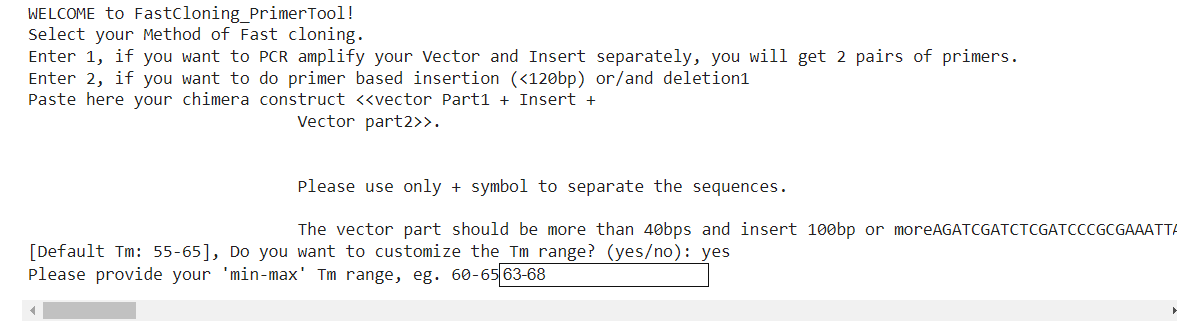


Press enter key.

**Sub step 5d: Save the designed primer as results in a text fil.**

You will see the next windows open to ask to save your results. Also result will be visible below script output space.


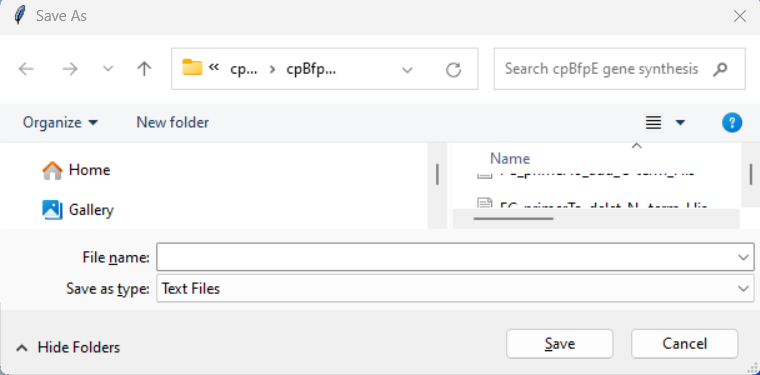


Your results will be saved as Text file at your defined locations.

**See example results in supplementary file S6 and S7.**

**Similar steps will be needed to follow for FC_class 2 primer designing, but the sequence input format will be different. See the manuscript for the details.**
