# Supplementary material for: Revolutionizing Molecular cloning: Introducing FastCloneAssist, a Streamlined Python tool for optimizing primer design in restriction & ligation-independent PCR cloning
Source: PLoS One. 2025 Mar 13;20(3):e0306950. doi: 10.1371/journal.pone.0306950 (PMC11906075; doi:10.1371/journal.pone.0306950)
Supplement: S7 File — (DOCX) [file pone.0306950.s007.docx]

**Example Sequence and input format:**

**Class 1: fast cloning to create a chimera using vector DNA and an insert sequence.**

**a)**


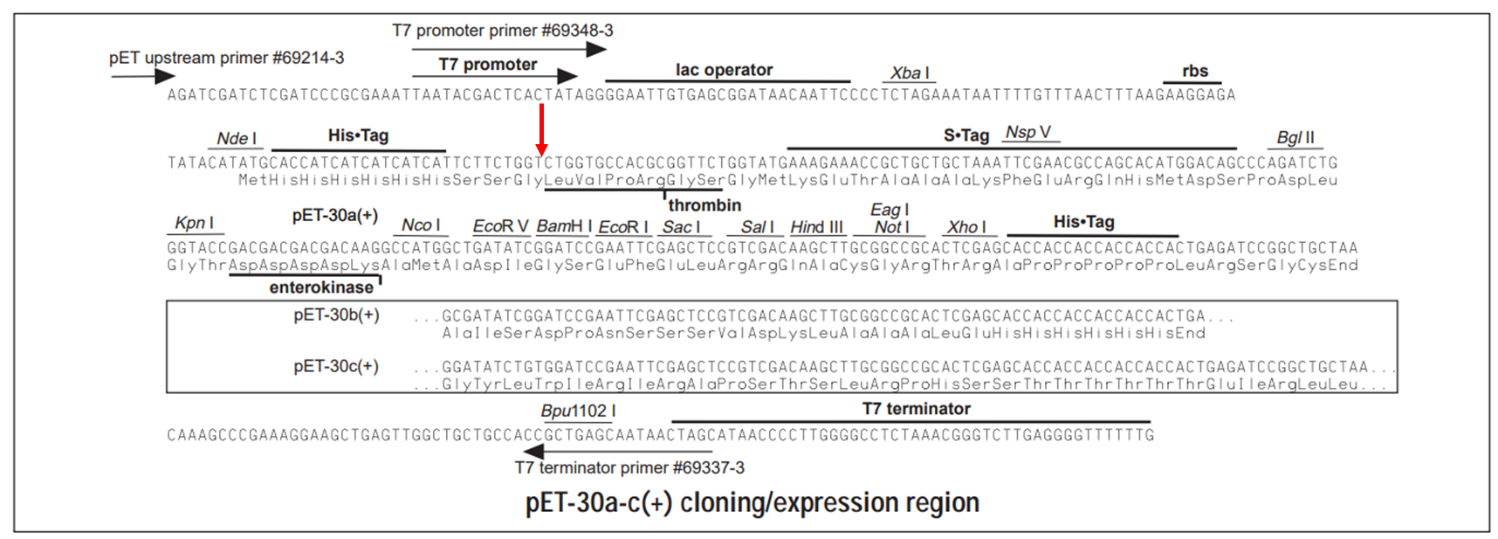


b)


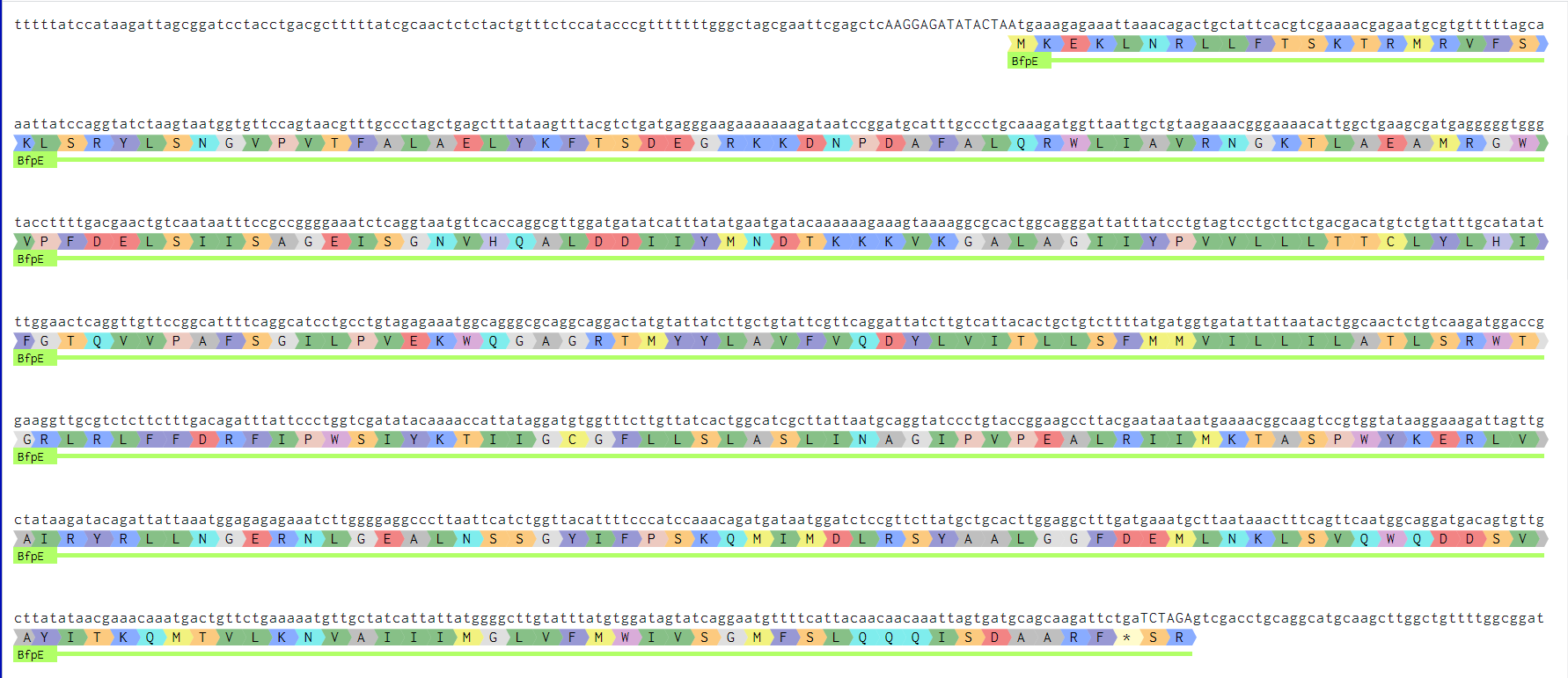


**Fig S1: a)** Example vector map. Red arrow indicates insertion site, just after glycine. b) Example sequence of insert gene, here BfpE highlighted in green, M to *.

Vector part 1 sequence (>40bp): sequences from left side of insertion point.

AGATCGATCTCGATCCCGCGAAATTAATACGACTCACTATAGGGGAATTGTGAGCGGATAACAATTCCCCTCTAGAAATAATTTTGTTTAACTTTAAGAAGGAGATATACATATGCACCATCATCATCATCATTCTTCTGGT

Vector part 2 sequence (>40bp): sequences from right side of insertion point.

CTGGTGCCACGCGGTTCTGGTATGAAAGAAACCGCTGCTGCTAAATTCGAACGCCAGCACATGGACAGCCCAGATCTGGGTACCGACGACGACGACAAGGCCATGGCTGATATCGGATCCGAATTCGAGCTCCGTCGACAAGCTTGCGGC

Insert DNA sequence:

atgaaagagaaattaaacagactgctattcacgtcgaaaacgagaatgcgtgtttttagcaaattatccaggtatctaagtaatggtgttccagtaacgtttgccctagctgagctttataagtttacgtctgatgagggaagaaaaaaagataatccggatgcatttgccctgcaaagatggttaattgctgtaagaaacgggaaaacattggctgaagcgatgagggggtgggtaccttttgacgaactgtcaataatttccgccggggaaatctcaggtaatgttcaccaggcgttggatgatatcatttatatgaatgatacaaaaaagaaagtaaaaggcgcactggcagggattatttatcctgtagtcctgcttctgacgacatgtctgtatttgcatatatttggaactcaggttgttccggcattttcaggcatcctgcctgtagagaaatggcagggcgcaggcaggactatgtattatcttgctgtattcgttcaggattatcttgtcattacactgctgtcttttatgatggtgatattattaatactggcaactctgtcaagatggaccggaaggttgcgtctcttctttgacagatttattccctggtcgatatacaaaaccattataggatgtggtttcttgttatcactggcatcgcttattaatgcaggtatccctgtaccggaagccttacgaataataatgaaaacggcaagtccgtggtataaggaaagattagttgctataagatacagattattaaatggagagagaaatcttggggaggcccttaattcatctggttacattttcccatccaaacagatgataatggatctccgttcttatgctgcacttggaggctttgatgaaatgcttaataaactttcagttcaatggcaggatgacagtgttgcttatataacgaaacaaatgactgttctgaaaaatgttgctatcattattatggggcttgtatttatgtggatagtatcaggaatgttttcattacaacaacaaattagtgatgcagcaagattctga

Organize input sequence for primer designing as; Vector part 1+ Insert + Vector part 2:

AGATCGATCTCGATCCCGCGAAATTAATACGACTCACTATAGGGGAATTGTGAGCGGATAACAATTCCCCTCTAGAAATAATTTTGTTTAACTTTAAGAAGGAGATATACATATGCACCATCATCATCATCATTCTTCTGGT **+** atgaaagagaaattaaacagactgctattcacgtcgaaaacgagaatgcgtgtttttagcaaattatccaggtatctaagtaatggtgttccagtaacgtttgccctagctgagctttataagtttacgtctgatgagggaagaaaaaaagataatccggatgcatttgccctgcaaagatggttaattgctgtaagaaacgggaaaacattggctgaagcgatgagggggtgggtaccttttgacgaactgtcaataatttccgccggggaaatctcaggtaatgttcaccaggcgttggatgatatcatttatatgaatgatacaaaaaagaaagtaaaaggcgcactggcagggattatttatcctgtagtcctgcttctgacgacatgtctgtatttgcatatatttggaactcaggttgttccggcattttcaggcatcctgcctgtagagaaatggcagggcgcaggcaggactatgtattatcttgctgtattcgttcaggattatcttgtcattacactgctgtcttttatgatggtgatattattaatactggcaactctgtcaagatggaccggaaggttgcgtctcttctttgacagatttattccctggtcgatatacaaaaccattataggatgtggtttcttgttatcactggcatcgcttattaatgcaggtatccctgtaccggaagccttacgaataataatgaaaacggcaagtccgtggtataaggaaagattagttgctataagatacagattattaaatggagagagaaatcttggggaggcccttaattcatctggttacattttcccatccaaacagatgataatggatctccgttcttatgctgcacttggaggctttgatgaaatgcttaataaactttcagttcaatggcaggatgacagtgttgcttatataacgaaacaaatgactgttctgaaaaatgttgctatcattattatggggcttgtatttatgtggatagtatcaggaatgttttcattacaacaacaaattagtgatgcagcaagattctga **+** CTGGTGCCACGCGGTTCTGGTATGAAAGAAACCGCTGCTGCTAAATTCGAACGCCAGCACATGGACAGCCCAGATCTGGGTACCGACGACGACGACAAGGCCATGGCTGATATCGGATCCGAATTCGAGCTCCGTCGACAAGCTTGCGGC

**Class 1: fast cloning to create a chimera using a vector DNA and an insert sequence after deleting a certain region from the vector DNA.**

**
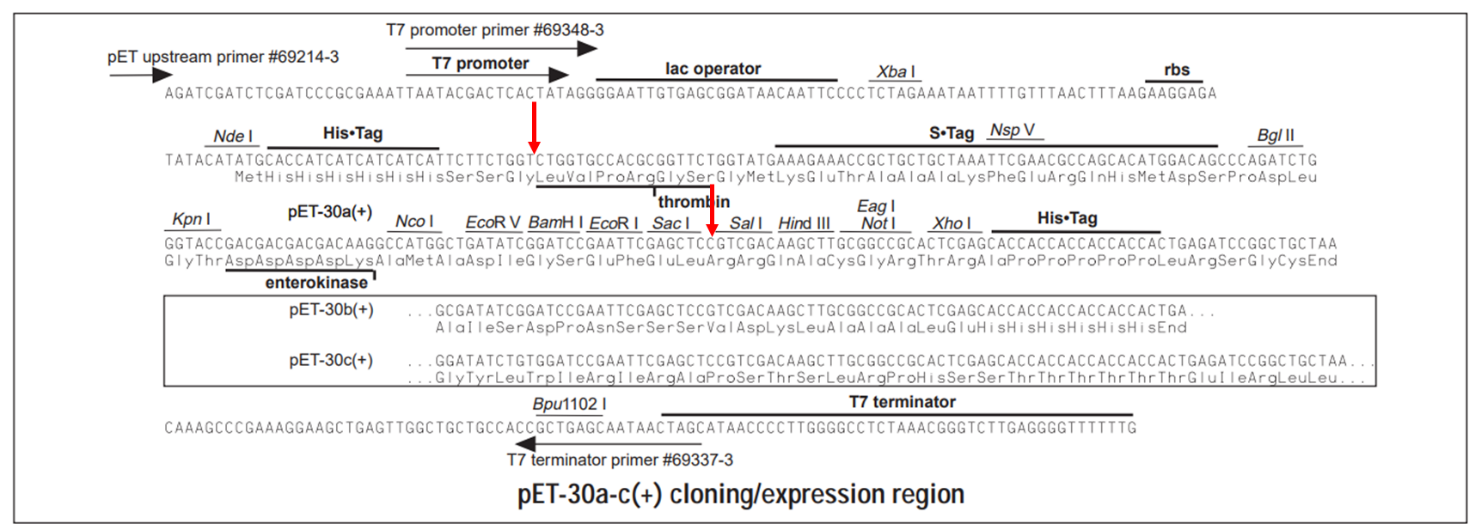
**

**Fig S2.** Example to insertion by deleting some region of vector. Here, we want to insert the same BfpE at pointed arrow to keep the His tag and end he sequence by *Sal*I restriction site.

Vector part 1 sequence (>40bp): sequences from left side of the first insertion point.

AGATCGATCTCGATCCCGCGAAATTAATACGACTCACTATAGGGGAATTGTGAGCGGATAACAATTCCCCTCTAGAAATAATTTTGTTTAACTTTAAGAAGGAGATATACATATGCACCATCATCATCATCATTCTTCTGGT

Vector part 2 sequence (>40bp): sequences from right side of the second insertion point.

GTCGACAAGCTTGCGGCCGCACTCGAGCACCACCACCACCACCACTGAGATCCGGCTGCTAA

**Insert DNA: Same as above.**

Organize input sequence for primer designing as; Vector part 1+ Insert + Vector part 2:

AGATCGATCTCGATCCCGCGAAATTAATACGACTCACTATAGGGGAATTGTGAGCGGATAACAATTCCCCTCTAGAAATAATTTTGTTTAACTTTAAGAAGGAGATATACATATGCACCATCATCATCATCATTCTTCTGGT **+** atgaaagagaaattaaacagactgctattcacgtcgaaaacgagaatgcgtgtttttagcaaattatccaggtatctaagtaatggtgttccagtaacgtttgccctagctgagctttataagtttacgtctgatgagggaagaaaaaaagataatccggatgcatttgccctgcaaagatggttaattgctgtaagaaacgggaaaacattggctgaagcgatgagggggtgggtaccttttgacgaactgtcaataatttccgccggggaaatctcaggtaatgttcaccaggcgttggatgatatcatttatatgaatgatacaaaaaagaaagtaaaaggcgcactggcagggattatttatcctgtagtcctgcttctgacgacatgtctgtatttgcatatatttggaactcaggttgttccggcattttcaggcatcctgcctgtagagaaatggcagggcgcaggcaggactatgtattatcttgctgtattcgttcaggattatcttgtcattacactgctgtcttttatgatggtgatattattaatactggcaactctgtcaagatggaccggaaggttgcgtctcttctttgacagatttattccctggtcgatatacaaaaccattataggatgtggtttcttgttatcactggcatcgcttattaatgcaggtatccctgtaccggaagccttacgaataataatgaaaacggcaagtccgtggtataaggaaagattagttgctataagatacagattattaaatggagagagaaatcttggggaggcccttaattcatctggttacattttcccatccaaacagatgataatggatctccgttcttatgctgcacttggaggctttgatgaaatgcttaataaactttcagttcaatggcaggatgacagtgttgcttatataacgaaacaaatgactgttctgaaaaatgttgctatcattattatggggcttgtatttatgtggatagtatcaggaatgttttcattacaacaacaaattagtgatgcagcaagattctga **+** GTCGACAAGCTTGCGGCCGCACTCGAGCACCACCACCACCACCACTGAGATCCGGCTGCTAA

**Class 2: Fast cloning to create a deletion, insertion, or combination of the two.**

1. **Deletion.**


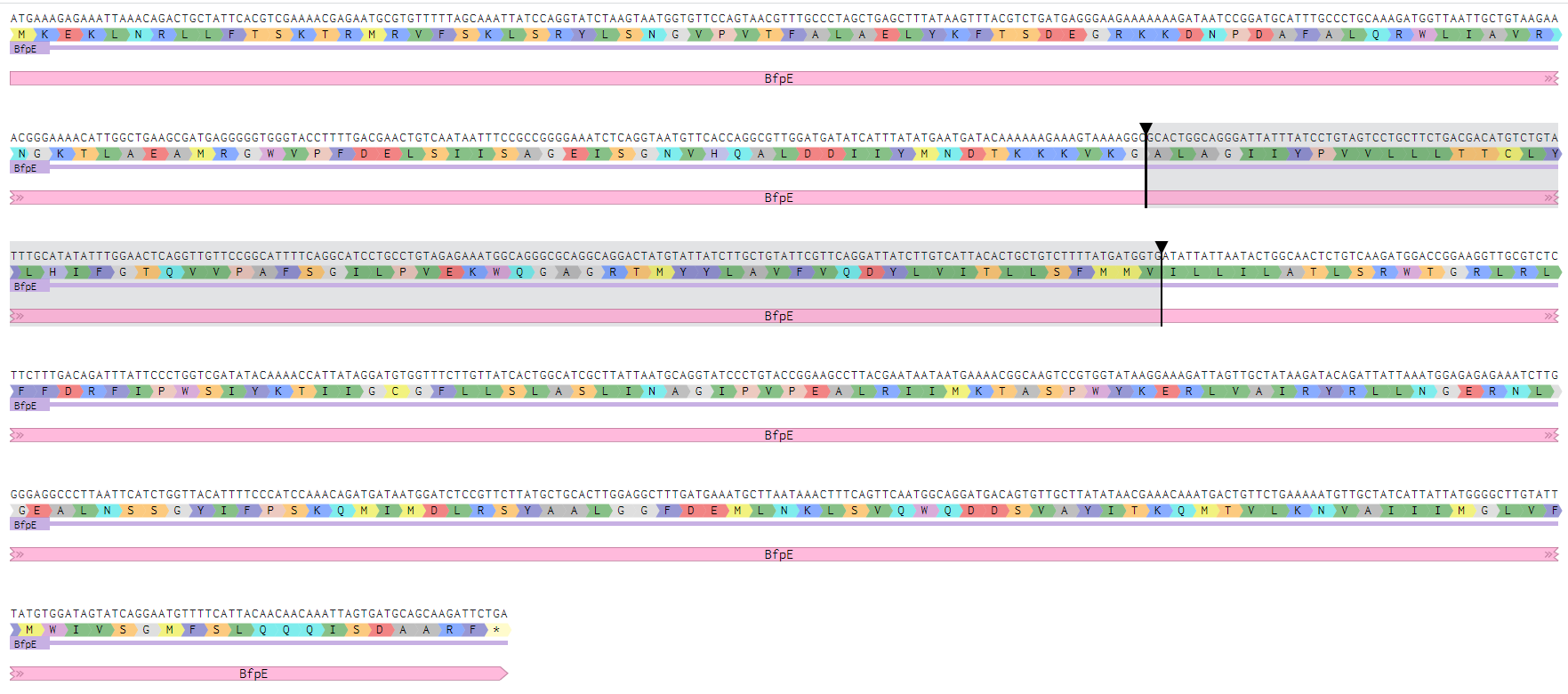


Fig 3S. I this example we want to delete the gray highlighted DNA sequence which is a transmembrane domain.

Input sequence format for this deletion will be:

ATGAAAGAGAAATTAAACAGACTGCTATTCACGTCGAAAACGAGAATGCGTGTTTTTAGCAAATTATCCAGGTATCTAAGTAATGGTGTTCCAGTAACGTTTGCCCTAGCTGAGCTTTATAAGTTTACGTCTGATGAGGGAAGAAAAAAAGATAATCCGGATGCATTTGCCCTGCAAAGATGGTTAATTGCTGTAAGAAACGGGAAAACATTGGCTGAAGCGATGAGGGGGTGGGTACCTTTTGACGAACTGTCAATAATTTCCGCCGGGGAAATCTCAGGTAATGTTCACCAGGCGTTGGATGATATCATTTATATGAATGATACAAAAAAGAAAGTAAAAGGC *

GCACTGGCAGGGATTATTTATCCTGTAGTCCTGCTTCTGACGACATGTCTGTATTTGCATATATTTGGAACTCAGGTTGTTCCGGCATTTTCAGGCATCCTGCCTGTAGAGAAATGGCAGGGCGCAGGCAGGACTATGTATTATCTTGCTGTATTCGTTCAGGATTATCTTGTCATTACACTGCTGTCTTTTATGATGGTG *

ATATTATTAATACTGGCAACTCTGTCAAGATGGACCGGAAGGTTGCGTCTCTTCTTTGACAGATTTATTCCCTGGTCGATATACAAAACCATTATAGGATGTGGTTTCTTGTTATCACTGGCATCGCTTATTAATGCAGGTATCCCTGTACCGGAAGCCTTACGAATAATAATGAAAACGGCAAGTCCGTGGTATAAGGAAAGATTAGTTGCTATAAGATACAGATTATTAAATGGAGAGAGAAATCTTGGGGAGGCCCTTAATTCATCTGGTTACATTTTCCCATCCAAACAGATGATAATGGATCTCCGTTCTTATGCTGCACTTGGAGGCTTTGATGAAATGCTTAATAAACTTTCAGTTCAATGGCAGGATGACAGTGTTGCTTATATAACGAAACAAATGACTGTTCTGAAAAATGTTGCTATCATTATTATGGGGCTTGTATTTATGTGGATAGTATCAGGAATGTTTTCATTACAACAACAAATTAGTGATGCAGCAAGATTCTGA *

The gray is deletion sequence marked my * symbol. The end of sequence also mut get marked by * symbol. Note: FastCloneAssist take care of gaps by itself.

1. **Insertion of a sequence**


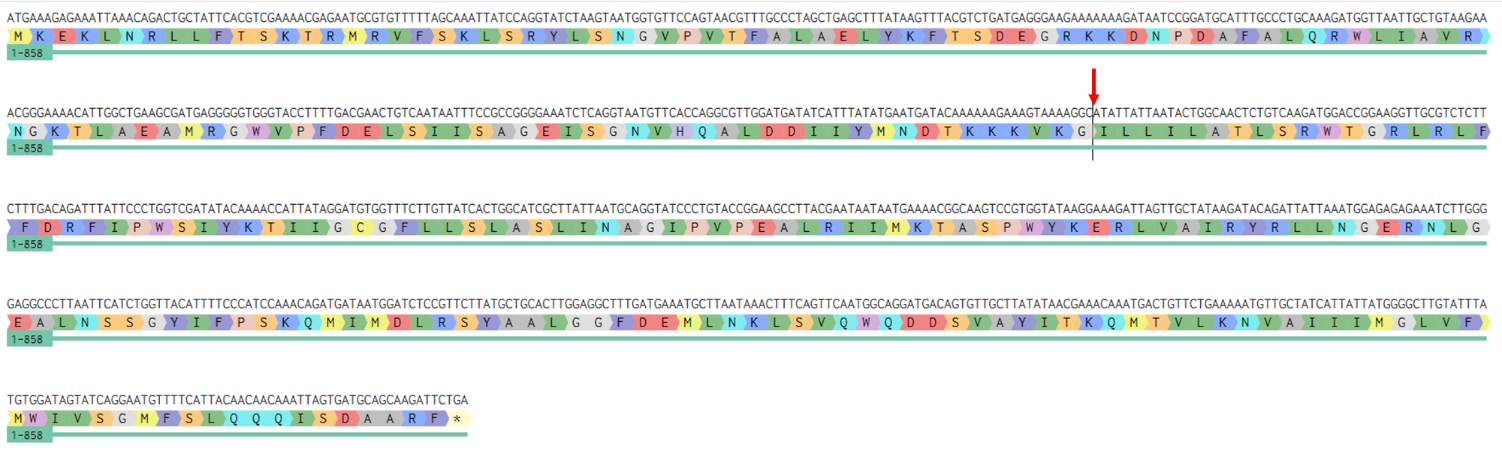


Fig 4S: Let assume we want to insert a small fluorophore tag (FLAsH tag) at the red arrow just after Gly.

The input sequence format for this class 2 primer designing will be.

ATGAAAGAGAAATTAAACAGACTGCTATTCACGTCGAAAACGAGAATGCGTGTTTTTAGCAAATTATCCAGGTATCTAAGTAATGGTGTTCCAGTAACGTTTGCCCTAGCTGAGCTTTATAAGTTTACGTCTGATGAGGGAAGAAAAAAAGATAATCCGGATGCATTTGCCCTGCAAAGATGGTTAATTGCTGTAAGAAACGGGAAAACATTGGCTGAAGCGATGAGGGGGTGGGTACCTTTTGACGAACTGTCAATAATTTCCGCCGGGGAAATCTCAGGTAATGTTCACCAGGCGTTGGATGATATCATTTATATGAATGATACAAAAAAGAAAGTAAAAGGC**ATATTATTAATACTGGCAACTCTGTCAAGATGGACCGGAAGGTTGCGTCTCTTCTTTGACAGATTTATTCCCTGGTCGATATACAAAACCATTATAGGATGTGGTTTCTTGTTATCACTGGCATCGCTTATTAATGCAGGTATCCCTGTACCGGAAGCCTTACGAATAATAATGAAAACGGCAAGTCCGTGGTATAAGGAAAGATTAGTTGCTATAAGATACAGATTATTAAATGGAGAGAGAAATCTTGGGGAGGCCCTTAATTCATCTGGTTACATTTTCCCATCCAAACAGATGATAATGGATCTCCGTTCTTATGCTGCACTTGGAGGCTTTGATGAAATGCTTAATAAACTTTCAGTTCAATGGCAGGATGACAGTGTTGCTTATATAACGAAACAAATGACTGTTCTGAAAAATGTTGCTATCATTATTATGGGGCTTGTATTTATGTGGATAGTATCAGGAATGTTTTCATTACAACAACAAATTAGTGATGCAGCAAGATTCTGA* TGTTGCCCGGGCTGCTGT

Note: The gray highlighted sequence is DNA sequence for FLAsH-tag (CCPGCC).

1. **Deletion and Insertion of a sequence**


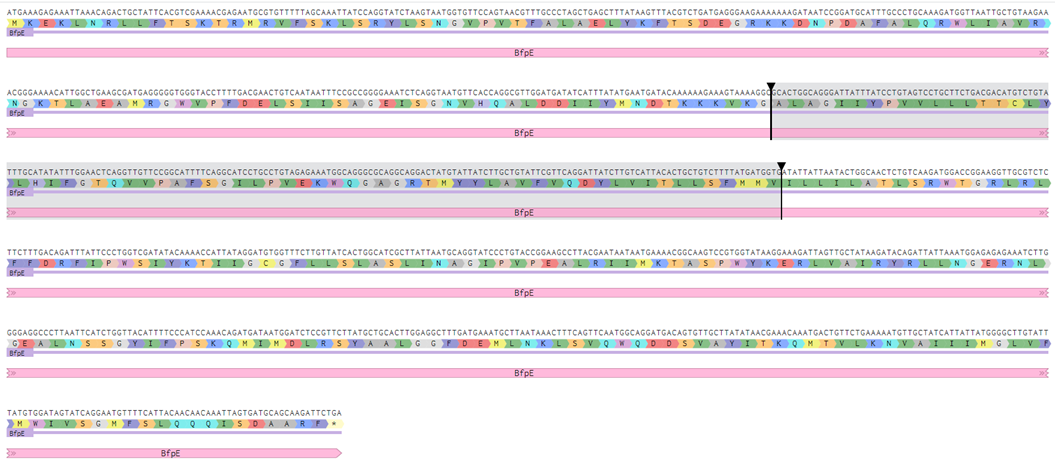


Fig S5: Lets assume here we want to delete the gray highlighted sequence and insert a FLAsH tag sequence there. The input sequence format will be.

ATGAAAGAGAAATTAAACAGACTGCTATTCACGTCGAAAACGAGAATGCGTGTTTTTAGCAAATTATCCAGGTATCTAAGTAATGGTGTTCCAGTAACGTTTGCCCTAGCTGAGCTTTATAAGTTTACGTCTGATGAGGGAAGAAAAAAAGATAATCCGGATGCATTTGCCCTGCAAAGATGGTTAATTGCTGTAAGAAACGGGAAAACATTGGCTGAAGCGATGAGGGGGTGGGTACCTTTTGACGAACTGTCAATAATTTCCGCCGGGGAAATCTCAGGTAATGTTCACCAGGCGTTGGATGATATCATTTATATGAATGATACAAAAAAGAAAGTAAAAGGC *

GCACTGGCAGGGATTATTTATCCTGTAGTCCTGCTTCTGACGACATGTCTGTATTTGCATATATTTGGAACTCAGGTTGTTCCGGCATTTTCAGGCATCCTGCCTGTAGAGAAATGGCAGGGCGCAGGCAGGACTATGTATTATCTTGCTGTATTCGTTCAGGATTATCTTGTCATTACACTGCTGTCTTTTATGATGGTG *

ATATTATTAATACTGGCAACTCTGTCAAGATGGACCGGAAGGTTGCGTCTCTTCTTTGACAGATTTATTCCCTGGTCGATATACAAAACCATTATAGGATGTGGTTTCTTGTTATCACTGGCATCGCTTATTAATGCAGGTATCCCTGTACCGGAAGCCTTACGAATAATAATGAAAACGGCAAGTCCGTGGTATAAGGAAAGATTAGTTGCTATAAGATACAGATTATTAAATGGAGAGAGAAATCTTGGGGAGGCCCTTAATTCATCTGGTTACATTTTCCCATCCAAACAGATGATAATGGATCTCCGTTCTTATGCTGCACTTGGAGGCTTTGATGAAATGCTTAATAAACTTTCAGTTCAATGGCAGGATGACAGTGTTGCTTATATAACGAAACAAATGACTGTTCTGAAAAATGTTGCTATCATTATTATGGGGCTTGTATTTATGTGGATAGTATCAGGAATGTTTTCATTACAACAACAAATTAGTGATGCAGCAAGATTCTGA * TGTTGCCCGGGCTGCTGT
