# Supplementary material for: Revolutionizing Molecular cloning: Introducing FastCloneAssist, a Streamlined Python tool for optimizing primer design in restriction & ligation-independent PCR cloning
Source: PLoS One. 2025 Mar 13;20(3):e0306950. doi: 10.1371/journal.pone.0306950 (PMC11906075; doi:10.1371/journal.pone.0306950)
Supplement: Attachment — Submitted filename: PLOS-review.docx [file pone.0306950.s008.docx]

The manuscript is written well in English.

Some figures can be included to show the tool and its response.

Comparison of time wrt tool’s operation like insert delete with traditional methods can be included.
