# Supplementary material for: Revolutionizing Molecular cloning: Introducing FastCloneAssist, a Streamlined Python tool for optimizing primer design in restriction & ligation-independent PCR cloning
Source: PLoS One. 2025 Mar 13;20(3):e0306950. doi: 10.1371/journal.pone.0306950 (PMC11906075; doi:10.1371/journal.pone.0306950)
Supplement: Attachment — Submitted filename: Response to Reviewers.docx [file pone.0306950.s009.docx]

School of Medicine

Virginia Commonwealth University School of Medicine

Sanger Hall, Room 1-055C

1101 E. Marshall St.

Richmond, VA 23298-5048

January 3, 2025

Mojtaba Kordrostami, Ph.D.

Academic Editor

PLOS ONE

Re.: PONE-D-24-21943

Dear Dr. Kordrostami,

Dr. Singh and I are grateful for your efforts in shepherding the above-referenced manuscript through initial review. As requested, below is our point-by-point response to the reviewer comments. Line numbers refer to the document with track changes.

**Comments in the file labelled PLOS-review.docx:**

1. **The manuscript is written well in English**.

No response required.

2. **Some figures can be included to show the tool and its response.**

We modified Figure 2 to include an additional image of the tool interface and an easy guide. See also lines 206-210.

3. **Comparison of time wrt tool’s operation like insert delete with traditional methods can be included.**

We appreciate your suggestion regarding a comparison of time efficiency. While a precise comparison between tool-based and traditional manual methods is challenging, we provided additional detail describing the advantages. Please see line numbers 223-233.

**Review comments to the author by Dr. Chattopadhyay:**

**Reviewer #1: The authors have meticulously performed the experimental procedure to design primers for PCR based Fast Cloning; but some issues have to be addressed before acceptance.**

**1. What is the utility of this web tool for designing primers for different length of inserts and different size of vectors. As the size is one of the key factors for cloning.**

We added a paragraph (lines 212-217) that specifies that success using the tool is dependent on the underlying PCR, including parameters such as the error rate, fidelity, and the maximum length of amplifiable DNA associated with the DNA polymerase used.

**2. The manuscript would be more attractive if authors provided some data on success rate in cloning using these primers.**

We modified a sentence in the case study section to highlight the success rate. Please see line number 273.

**3. Is it possible to do the location specific insertion, deletion and mutation using these primers, without changing the reading frame.**

The introductory paragraph was modified for enhanced clarity so that readers will understand that the method does indeed make this possible. See lines 48-52.

**4. I would also suggest to improve the picture quality.**

Original figures 1 and 2 (2 now 3) were modified to improve clarity and a new figure (2) was added.

**Response to the Editor’s comments in email:**

**Journal Requirements:**

1. **When submitting your revision, we need you to address these additional requirements.

   Please ensure that your manuscript meets PLOS ONE's style requirements, including those for file naming. The PLOS ONE style templates can be found at**
   [**https://journals.plos.org/plosone/s/file?id=wjVg/PLOSOne_formatting_sample_main_body.pdf**](https://urldefense.com/v3/__https:/track.editorialmanager.com/CL0/https:*2F*2Fjournals.plos.org*2Fplosone*2Fs*2Ffile*3Fid=wjVg*2FPLOSOne_formatting_sample_main_body.pdf/1/010f0193a8aadcc8-88e94278-6114-4f8a-a3e5-ad96e096ea96-000000/qj-nnKQ2pGtWEWMf4km3ewBoV5SXznjOpludAdG49sk=188__;JSUlJSUlJQ!!JqxBPMk!kMz7QxXwxliL9MAnSwKn_goV4vTQgEGzyaL5uT5MvurtcS8RtVmfk9sds1U0CoGi7v0G-vVdCuP0KDvMeEmqtd8$) **and**
   [**https://journals.plos.org/plosone/s/file?id=ba62/PLOSOne_formatting_sample_title_authors_affiliations.pdf**](https://urldefense.com/v3/__https:/track.editorialmanager.com/CL0/https:*2F*2Fjournals.plos.org*2Fplosone*2Fs*2Ffile*3Fid=ba62*2FPLOSOne_formatting_sample_title_authors_affiliations.pdf/1/010f0193a8aadcc8-88e94278-6114-4f8a-a3e5-ad96e096ea96-000000/ATz3RpnfVtrryZbxbQVvuOtagvOBfmzqGJUfFQZ9HxE=188__;JSUlJSUlJQ!!JqxBPMk!kMz7QxXwxliL9MAnSwKn_goV4vTQgEGzyaL5uT5MvurtcS8RtVmfk9sds1U0CoGi7v0G-vVdCuP0KDvMf17L6Bw$)

We reviewed the PLOS ONE style requirements and made several changes.

Lines 166-167: “Supplementary file” replaced by “S1 File”.

Lines 190-191, 234-236, 290, 302, 308, 311, supplementary files were renamed from text and folder as specified in the instructions.

**2. We note that the grant information you provided in the ‘Funding Information’ and ‘Financial Disclosure’ sections do not match.

When you resubmit, please ensure that you provide the correct grant numbers for the awards you received for your study in the ‘Funding Information’ section.**

We modified the sentence to indicate that there is no award number.

**3. Please expand the acronym "VCU" (as indicated in your financial disclosure) so that it states the name of your funders in full.
This information should be included in your cover letter; we will change the online submission form on your behalf.**

Modified as requested

On behalf of Dr. Singh and myself, I am pleased to resubmit this manuscript and look forward to your final decision.

Sincerely yours,

Michael S. Donnenberg, MD

Professor of Internal Medicine, Microbiology & Immunology, and Biochemistry & Molecular Biology

Senior Associate Dean for Research and Research Training

Director, Medical Scientist Training Program

Virginia Commonwealth University School of Medicine
